# Invariable Ribosome Stoichiometry During Murine Erythroid Differentiation: Implications for Understanding Ribosomopathies

**DOI:** 10.3389/fmolb.2022.805541

**Published:** 2022-02-03

**Authors:** Christos I. Papagiannopoulos, Konstantinos A. Kyritsis, Konstantina Psatha, Dimitra Mavridou, Fani Chatzopoulou, Georgia Orfanoudaki, Michalis Aivaliotis, Ioannis S. Vizirianakis

**Affiliations:** ^1^ Laboratory of Pharmacology, School of Pharmacy, Aristotle University of Thessaloniki, Thessaloniki, Greece; ^2^ Functional Proteomics and Systems Biology (FunPATh)—Center for Interdisciplinary Research and Innovation (CIRI-AUTH), Thessaloniki, Greece; ^3^ Laboratory of Biochemistry, School of Medicine, Faculty of Health Sciences, Aristotle University of Thessaloniki, Thessaloniki, Greece; ^4^ Basic and Translational Research Unit, Special Unit for Biomedical Research and Education, School of Medicine, Aristotle University of Thessaloniki, Thessaloniki, Greece; ^5^ Institute of Molecular Biology and Biotechnology, Foundation of Research and Technology, Heraklion, Greece; ^6^ Laboratory of Microbiology, School of Medicine, Aristotle University of Thessaloniki, Thessaloniki, Greece; ^7^ Department of Life and Health Sciences, University of Nicosia, Nicosia, Cyprus

**Keywords:** ribosomopathies, ribosomal proteins, erythropoiesis, proteomics, mass spectrometry

## Abstract

Heterogeneity of the main ribosomal composition represents an emerging, yet debatable, mechanism of gene expression regulation with a purported role in ribosomopathies, a group of disorders caused by mutations in ribosomal protein genes (RPs). Ribosomopathies, mysteriously relate with tissue-specific symptoms (mainly anemia and cancer predisposition), despite the ubiquitous expression and necessity of the associated RPs. An outstanding question that may shed light into disease pathogenicity and provide potential pharmacological interventions, is whether and how the ribosomal composition is modified during, the highly affected by RP mutations, process of erythroid differentiation. To address this issue, we analyzed ribosome stoichiometry using an established model of erythroid differentiation, through sucrose gradient ultracentrifugation and quantitative proteomics. We found that differentiation associates with an extensive reprogramming of the overall ribosomal levels, characterized by an increase in monosomes and a decrease in polysomes. However, by calculating a stoichiometry score for each independent ribosomal protein, we found that the main ribosomal architecture remained invariable between immature and differentiated cells. In total, none of the 78 Ribosomal Proteins (RPs- 74 core RPs, Rack1, Fau and 2 paralogs) detected was statistically different between the samples. This data was further verified through antibody-mediated quantification of 6 representative RPs. Moreover, bioinformatic analysis of whole cell proteomic data derived out of 4 additional models of erythropoiesis revealed that RPs were co-regulated across these cell types, too. In conclusion, ribosomes maintain an invariant protein stoichiometry during differentiation, thus excluding ribosome heterogeneity from a potential mechanism of toxicity in ribosomopathies and other erythroid disorders.

## Introduction

Ribosomes constitute the main macromolecular machines that catalyze protein synthesis within the cells of all domains of life. Each ribosome is assembled by the same set of 80 Ribosomal proteins (RPs) and 4 ribosomal RNAs (rRNAs) which structurally associate into two subunits, the 40S and the 60S (Montanaro et al., 2008; Wilson and Doudna Cate, 2012). The small subunit is responsible for the recognition and binding of ribosomes to cytosolic mRNAs, while the large subunit catalyzes peptide bond formation. This core ribosomal structure is highly conserved across bacteria, archaea, and eukaryotes (Bowman et al., 2020).

Recently however, it has been proposed that some ribosomal subgroups may demonstrate alterations that differentiate them from the typical ribosomal machinery. This kind of ribosome heterogeneity may arise through quantitative alterations of one or more RPs that disrupt the equimolar (1:1) ratio between the RPs in each ribosomal entity (Sauert et al., 2015; Guimaraes and Zavolan, 2016; Genuth and Barna, 2018; Sulima and Dinman, 2019; Li and Wang, 2020). Experimentally, ribosome heterogeneity has been identified in diverse organisms by analyzing purified ribosomal populations using quantitative proteomics. Heterogeneous ribosomes were, indeed, identified in mouse embryonic stem cells (Shi et al., 2017), yeast (Slavov et al., 2015), colon cancer (Kimura et al., 2010) and muscle cells (Chaillou et al., 2016) implicating several RPs including RPL38, RPL25, RPL10a and RPL3. By a similar mechanism, additional alterations, such as rRNA modifications, changes in ribosome associated proteins (Raps) and post-translational modifications can also give rise to heterogeneous ribosomal subtypes in a given cell type (Sauert et al., 2015; Sulima and Dinman, 2019). Ostensibly heterogeneous ribosomes may show increased affinity for selected 5-untraslated regions in transcripts and thus be specialized to preferentially translate distinct mRNA subgroups (Xue and Barna, 2012).

On the contrary though, several studies contradict the hypothesis of ribosome heterogeneity and its potential implications in gene expression regulation. For example, the core ribosomal architecture (composed of 80 RPs) was found invariable in purified polysomes derived from 5 prostate cancer cell lines and mouse embryonic fibroblasts (Reschke et al., 2013). Similarly, a constant RPs stoichiometry was reported in a proteomic comparison between monosomes and polysomes across 3 different brain and liver tissues in old versus young mice (Amirbeigiarab et al., 2019). Also, a recent analysis from our group showed that the RP transcripts demonstrate a consistent expression landscape across 33 human tissues, with limited variation arising only from species idiosyncrasies and tissue-specific expression of RP paralogs (Kyritsis et al., 2020). This controversy suggests that heterogeneity of the main ribosomal composition may be a tissue- specific phenomenon rather than a widespread translation- regulating mechanism.

Perhaps the most relevant tissue to study the ribosomal composition and function is the erythroid lineage. Congenital mutations in a variety of RP genes disrupt ribosome homeostasis and result in the appearance of rare clinical syndromes, known as ribosomopathies (Narla and Ebert, 2010). Intriguingly, despite the fact that the mutated RPs are ubiquitously expressed across all tissues, ribosomopathies manifest with tissue-specific symptoms in patients, that typically relate to an inability to produce functional red blood cells (Narla and Ebert, 2010; Sakamoto and Narla, 2018; Farley-Barnes et al., 2019). This selective toxicity is evident in patients with Diamond-Blackfan Anemia (DBA), the most frequent ribosomopathy, who primarily suffer from anemia and cancer predisposition (Ellis and Gleizes, 2011; Amanatiadou et al., 2015; Ulirsch et al., 2018; Da Costa et al., 2020.

Mechanistically, RP mutations provoke a reduction in the levels of the encoded proteins and ostensibly can give rise to ribosomes with varying stoichiometry across tissues. It is possible that such heterogeneous ribosomes are critical for erythroid development and that mutations of ribosomal components disrupt this mechanism, hence leading to selective toxicity in the erythroid lineage (Genuth and Barna, 2018). This association creates the question of whether the main ribosomal composition (ie. the 80 ribosomal proteins) is modified during erythroid differentiation.

To address this issue, we performed a comprehensive analysis of ribosome regulation and stoichiometry during murine erythropoiesis. We utilized classical biochemical techniques for the isolation of ribosomal populations, combined with state-of-the art quantitative proteomics and bioinformatic methods. Firstly, we confirmed previous observations that the overall ribosome levels are altered dramatically during erythroid differentiation (Hensold et al., 1996). This change involves a decrease of the polysomal particles, accompanied by a significant increase in monosomes. Moreover, our proteomic analysis identified the same set of 78 RPs in all analyzed samples, but none of these RPs was quantitatively different between the samples. We validated this data using RP specific antibodies against 6 RPs in an immunoblot setting and, again, found no differentiation dependent change in their levels. Moreover, all RPs demonstrated significant positive correlation in their expression levels across 4 additional models of erythropoiesis, irrespective of the differentiation status. In conclusion, our analysis supports a model were the RP composition remains invariable during erythroid differentiation and any ribosome-mediated regulation may arise through changes in the overall ribosome levels or other mechanisms of heterogeneity (such as alterations in rRNA composition).

## Materials and Methods

### MEL Cell Culture and Induction of Differentiation

The established permanent cancer model MEL-745 (murine erythroleukemia FLC clone 745) represented the main cellular model of this work and it was handled in a way to maintain cells with high inducibility of erythroid differentiation. To this end, cells were diluted every 3 days with fresh medium and maintained at a concentration between 5 × 10^4^ and 5 × 10^5^ cells/ml. The MEL cells were obtained from Dr. C. Friend (Division of Cytology, The Sloan-Kettering Institute for Cancer Research, New York, NY, United States) and were grown in an incubator under standard conditions. Erythroid differentiation was induced by in culture treatment with 5 × 10^−3^ M HMBA dissolved in water (Marks and Rifkind, 1988; Vizirianakis and Tsiftsoglou, 1996; Tsiftsoglou et al., 2003a; Papagiannopoulos et al., 2021a).

### Cell Proliferation and Differentiation Assays

Cellular proliferation was determined using an optical microscope in a Neubauer counting chamber (Paul Marienfeld GmbH & Co. KG, Lauda-Königshofen, Germany). Moreover, when needed, cell death was assessed using the Trypan blue dye-exclusion method, essentially as previously described. Also, we utilized the benzidine–H_2_O_2_ assay to score erythroid differentiation of MEL cells after treatment with HMBA, directly in cultured cells. The assay was performed as previously described in (Vizirianakis et al., 2015; Papagiannopoulos et al., 2021b), by assessing at least 300 cells of each culture.

### Gradient Preparation and Ultracentrifugation

The generation of a linear sucrose gradient was done using a modification of the approach described in Luthe (1983). We, initially, prepared the independent 10% and 50% sucrose solution in the following buffer: 20 mM Tris, pH 7.5, 100 mM, NaCl, 15 mM MgCl_2_, 100 μg/ml cycloheximide. After filling ¼ of the tube (approximately 2.8 ml in a SW41Ti rotor tube) with the heavy 50% solution the tube was frozen at −80°C. Then a mixture of 2 parts of 50% with 1 part of 10% sucrose was applied on top, followed by a mixture of 50–10% 1:2. Finally, we applied the 10% sucrose for the rest of the volume of the tube. Between each step the solution was allowed to freeze at −80°C. One day before use the solutions were allowed to thaw at 4C overnight. Samples were ultracentrifuged at 30,000 rpm for 3 h in a SW41Ti rotor.

### Ribosome Fractionation

Approximately 1-2 × 10^7^ cells were harvested for ribosome fractionation and treated for 5 min with 100 μg/ml cycloheximide (Merck & Co., Inc., Kenilworth, NJ, United States). Cell lysis was achieved by exposure to mild detergent conditions after incubation with the following lysis buffer: 20 mM Tris pH 7.5, 150 mM NaCl, 15 mM MgCl2, 100 μg/ml cycloheximide, 1 mM DTT, 1% Triton X-100, 6% glycerol, DNase (DNase I, Amplification Grade, Merck & Co., Inc., Kenilworth, NJ, United States), protease and phosphatase Inhibitor (MS-SAFE, Merck & Co., Inc., Kenilworth, NJ, United States). To ensure efficient lysis samples were incubated on ice for 30 min. The samples were then subjected to two rounds of centrifugation: 1) for 5 min at 1,800x g, 4°C and 2) 100,00x g for 5 min at 4°C. Samples with equal values of absorption at 260 nm were ultracentrifuged at 30,000 rpm for 3 h in a SW41Ti rotor inside a 10–50% sucrose gradient.

### RNA Analysis of Sucrose Fractions

All gradients were split in either 12 or 16 fractions after ultracentrifugation and they were processed for analysis. To visualize the distribution of rRNAs along the sucrose gradient RNA was precipitated by mixing 1 volume of sample with equal volume of denaturing solution D (4 M guanidinium thiocyanate, 25 mM sodium citrate, 0.5% (w/v) sodium lauryl sarcosinate, 0.1 M ß-mercaptoethanol) followed by the addition of sodium acetate (pH 5.2, 0.25 M). To this mixture, 0.7 volumes of isopropanol were added, and the samples were left at −20°C overnight. The next day the mixture was centrifuged at maximum speed for 15 min at 4C to precipitate RNA. The precipitated RNA was finally separated in a 2% agarose gel, stained with EtBr and visualized under UV light.

### Western Blot Analysis

Approximately 10 μg of each sample was used for Western blot analysis. Protein quantification, gel preparation and electrophoresis were conducted as previously described (Papagiannopoulos et al., 2021b). Proteins were transferred to a PVDF membrane and blotted with primary antibodies over-night at 4°C and with secondary antibody for 1 h at room temperature. We used the following antibodies (From Santa Cruz Biotechnology, Dallas, TX, United States): Hemoglobin β/γ/δ/ε: sc-390668, Laminin-R: sc-74515, m-IgGκBP-HRP: sc-516102, RACK1: sc-17754, RPS6: sc-74459, RPS7: sc-377317, RPS17: sc-100835, RPS23: sc-100837, RPL4: sc-100838, RPS10: sc-515655, RPS19: sc-134779, α-Tubulin: sc-51503, β-Actin: sc-47778.

### Sample Preparation for Proteomics

Protein extraction out of sucrose fractions was performed by ultrafiltration using the Amicon^®^ Ultra-4 Centrifugal Filter Units (Merck & Co., Inc., Kenilworth, NJ, United States) with a membrane size of 3 kDa, according to the manufacturers’ protocol. Samples were prepared in electrophoresis sample buffer, boiled for 15 min, and loaded into a 12% separating gel. The gel was stained with Coomassie Brilliant Blue and processed with in-gel digestion exactly as described by Shevchenko et al. (2006). Briefly, each gel was sliced into 1 mm^3^ cubes and washed with 50 mM NH4HCO3: Acetonitrile (ACN) = 50:50 until discoloration was observed. The proteins were then reduced with 10 mM DTT (at 56°C for 1 h) and alkylated with 50 mM IAA (treated for 30 min at room temperature in the dark). Proteolysis was performed with trypsin solution (Promega, Madison, WI, United States) overnight at 37°C. The samples were then acidified with 0.1% TFA, lyophilized to near dryness, and finally resuspended 0.1% formic acid before LC-MS/MS analysis.

### Mass Spectrometry Analysis

For the MS analysis, we used pooled fractions 10–16 from the corresponding samples, purified from sucrose solution by filtration. The samples were sent to Creative Proteomics (Shirley, New York, United States) to conduct label-free quantitative proteomic analysis.

The nLC-ESI-MS/MS was performed in an Ultimate 3000 nano UHPLC system (ThermoFisher Scientific, United States) coupled with a Q Exactive HF mass spectrometer (Thermo Fisher Scientific, United States) and an ESI nanospray source.

The nanoLC analysis was performed using a trapping column: PepMap C18, 100 Å, 100 μm × 2 cm, 5 μm; and an analytical column: PepMap C18, 100 Å, 75 μm × 50 cm, 2 μm. Each sample was loaded equally (1 μg) on the nLC system. The mobile phase was A: 0.1% formic acid in water; B: 0.1% formic acid in 80% acetonitrile and the flow rate: 250 nl/min. LC linear gradient: from 2 to 8% buffer B in 3 min, from 8 to 20% buffer B in 40 min, from 20 to 40% buffer B in 28 min, then from 40 to 90% buffer B in 4 min.

The MS full scan was performed between 300–1,650 m/z at the resolution 60,000 at 200 m/z, the automatic gain control target for the full scan was set to 3e6. The MS/MS scan was operated in Top 20 mode using the following settings: resolution 15,000 at 200 m/z; automatic gain control target 1e5; maximum injection time 19 ms; normalized collision energy at 28%; isolation window of 1.4 Th; charge sate exclusion: unassigned, 1, >6; dynamic exclusion 30 s.

The resulted Raw MS files were analyzed and searched against mouse protein database based on the species of the samples using Maxquant (1.6.2.6). The parameters were set as follows: the protein modifications were carbamidomethylation (C) (fixed), oxidation (M) (variable); the enzyme specificity was set to trypsin; the precursor ion mass tolerance was set to 10 ppm, and MS/MS tolerance was 0.5 Da.

### Bioinformatic Data Analysis

The intensity values of RPs were scaled, by dividing with the sum of RP intensity values per sample, to represent the stoichiometry of RPs per sample and differentiation stage. Over-representation analyses of GO terms (Molecular Function; MF) for the discovery of statistically significant terms [False Discovery Rate (FDR) adjusted *p*-value < 0.05] were performed using clusterProfiler. Intensity values for RPs were retrieved from whole cell proteomics experiments of Gautier et al. (2020), for all types of erythroid cell models and differentiation stages and normalized to z-score.

## Results

### MEL Cells as a Model of Erythroid Differentiation

The MEL cell line represents an established *in- vitro* system in the research of fundamental mechanisms of the erythroid cell lineage. MEL cells, which are blocked at the proerythroblast stage of differentiation and demonstrate malignant characteristics, can be induced to differentiate by chemical compounds, such as Hexamethylene Bisacetamide (HMBA), and lose tumorigenicity (Vizirianakis et al., 1992; Tsiftsoglou et al., 2003a; Tsiftsoglou et al., 2003b). Treatment with HMBA initiates commitment to differentiation and induces drastic alterations that include: 1) escape from the malignant phenotype (Supplementary Figure S1A) 2) gradual size decrease (Supplementary Figure S1B), 3) expression of erythroid markers including synthesis of hemoglobin (Matragkou et al., 2008; Vizirianakis et al., 2015; Papagiannopoulos et al., 2021a). The most significant alterations take place during the first 48 h of the process, therefore we isolated samples within this time frame.

### Ribosome Fractionation Reveals an Extensive Reorganization of Ribosomes During Erythropoiesis

To study ribosome regulation in the MEL cell system, we utilized a ribosome fractionation protocol based on sucrose gradient (10–50%) ultracentrifugation. By this method we efficiently separated ribosomes from the rest of the cytoplasmic material, shown by the migration of Rps19 into the heavy fractions of the gradient, in contrast to b-tubulin which demonstrated limited mobility only until fraction 4 (Supplementary Figure S2A). Moreover, efficient ribosome fractionation was validated by RNA extraction and visualization of the 18S and 28S rRNAs through agarose gel electrophoresis and staining with EtBr (Supplementary Figure S2B). Also, EDTA treatment resulted in polysome dissociation and disappearance of ribosomes from the heavy fractions, as expected (Supplementary Figure S2B). In total, polysomes migrated to the heavy fractions of the gradient (fractions 10–16), monosomes stayed in the middle of the gradient (fractions 7–8), and free subunits were found in the beginning of the gradient (fractions 4–5, Figure 2B). Finally, SDS-PAGE electrophoresis of isolated polysomal proteins (pooled fractions 10–16) extracted out of four stages of MEL cell differentiation, revealed that polysomes are devoid of most cytosolic proteins (Supplementary Figure S3). Thus, our protocol efficiently resulted in ribosome fractionation and purification from most cytosolic proteins.

**FIGURE 1:**
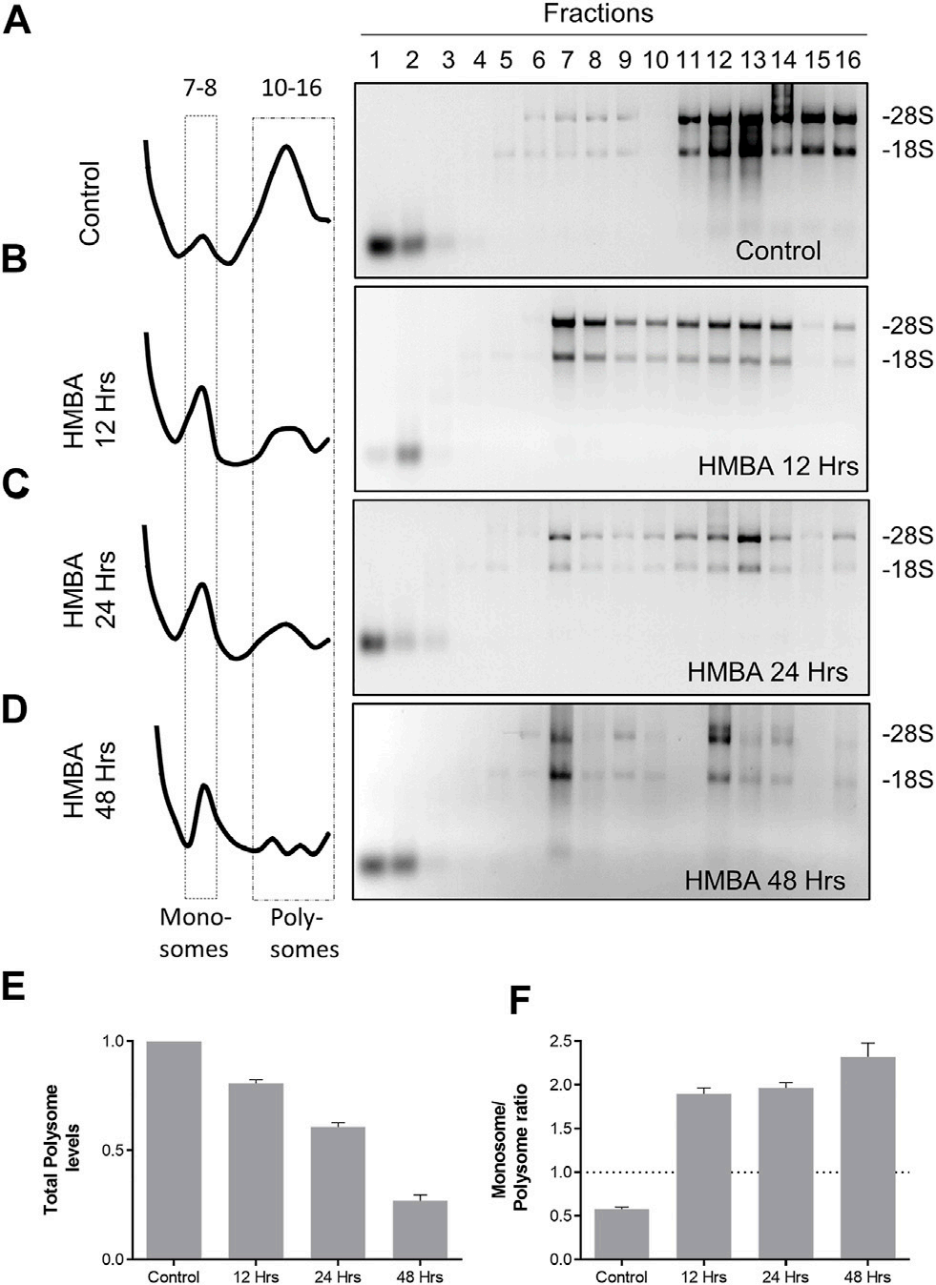
Regulation of ribosome levels during MEL differentiation. **(A–D)** Analysis of the total RNA material isolated from each fraction of the gradient for the corresponding samples (Control, HMBA 12 h, HMBA 24 h and HMBA 48 h treatment). Each sample was electrophorized in a 2% agarose gel, stained with Etbr and visualized under UV light (experiment was repeated at least 3 times). The left panel corresponds to the A260 profile of each gradient. **(E)** Alterations in the total polysome levels. Each bar corresponds to the average A260 value of fractions 10–16. **(F)** Monosome/polysome ratio for each sample. The ratio was calculated by dividing average fractions 5–6/average fractions 10–16.

Moreover, we determined the absorbance at 260 nm (A260) along the length of the gradient, to provide a quantification of the levels of ribosomal particles that populate each part of the gradient. By this method, we validated previous findings (Hensold et al., 1996) that MEL differentiation involves an extensive reorganization of ribosomes, characterized by a reduction of polysomes and increase of the monosomal particles (Figures 1A–D). These alterations already happened during 12 h of differentiation and continued to occur until the final stages (Figures 1E,F). Thus, by using a ribosome fractionation protocol we efficiently separated ribosomes from the rest of the cytoplasmic constituents and identified critical aspects of ribosomal regulation during MEL differentiation.

### Mass Spectrometry Analysis of Purified Polysomes

**FIGURE 2:**
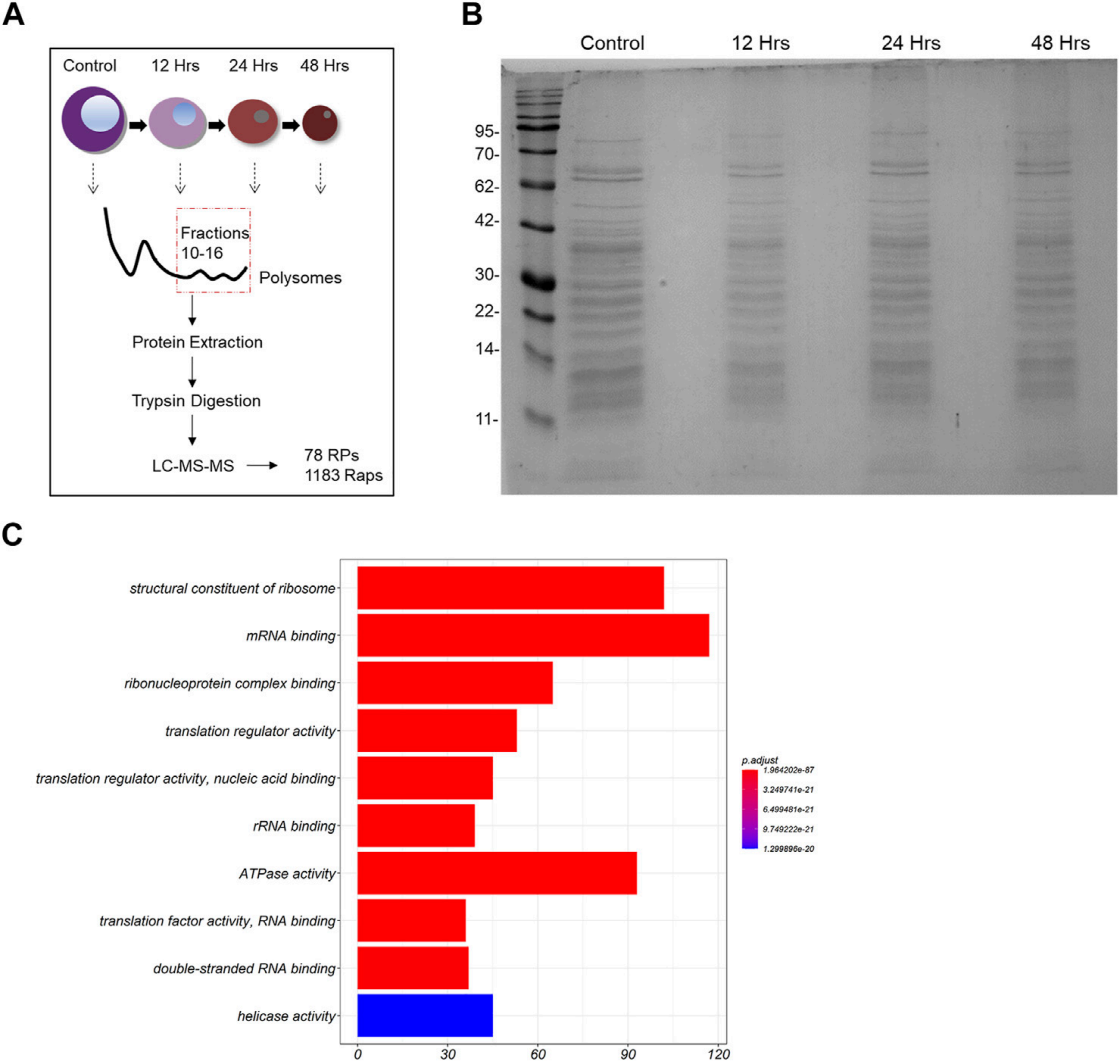
Analysis of the riboproteome in MEL cells. **(A)** Design of the study. **(B)** Total protein material was extracted from pooled fractions 10–16 by protein filtration. The proteins were then loaded on an SDS-PAGE gel, stained with blue silver staining and depicted. **(C)** Gene ontology (GO) Molecular Function (MF) terms enriched in the 1261 proteins (RPs and Raps) that are part of the untreated MEL cells’ ribo-proteome. The ten most statistically significant terms (FDR adjusted *p*-value < 0.05) are shown in the barplot (*y*-axis) along with the number associated proteins (*x*-axis). The over-representation analysis was performed using clusterProfiler.

Polysomes represent the most active ribosomal subtypes within eukaryotic cells (in comparison to monosomes and free subunits), therefore leading us to focus on the analysis of the polysomal composition across our samples of interest. For this reason, we extracted the total protein material out of the 10th until the 16th fraction of each sucrose gradient and pooled all fractions in one sample (graphical representation of the experiment is depicted in Figure 2A). We then loaded equal amounts of extracted proteins from 2 time points (control cells and 48 h of differentiation) on an SDS-PAGE gel. We noticed that the macroscopic examination of the stained gel revealed no apparent differences between the samples (Figure 2B). In total, 1,261 proteins met the requirements for quantitative comparison between the 2 samples (control and 48 h of differentiation) in 4 biological replicates of the experiment (the data generated through the MaxQuant analysis are given as 2 Supplementary Data Sheets S2–4). Moreover, we achieved high coverage of almost all RPs, as 75 of the 79 core RPs, Rack1 and 2 of the 8 RP paralogs were quantified in all samples. The missing RPs are either localized at the ribosomal surface and are likely lost during centrifugation, or after tryptic proteolysis they generated peptides that cannot be detected by this method. All quantified RPs were identified by at least 2 unique peptides. To examine the molecular functions that may be enriched in our dataset, we carried out gene ontology (GO) enrichment analysis of all 1,261 proteins. Importantly, we found that terms such as: “structural constituent of the ribosome”, “translation regulator activity” and “mRNA binding” are highly enriched in our protein group (Figure 2C). Moreover, RPs clustered together in a protein-protein interaction network and formed connections with ribosome associated proteins with varying functionality, including RNA-binding proteins and ribosome factors (Supplementary Image S1). Thus, we confirmed that our experimental pipeline indeed generated purified ribosomal particles from MEL cells.

**FIGURE 3:**
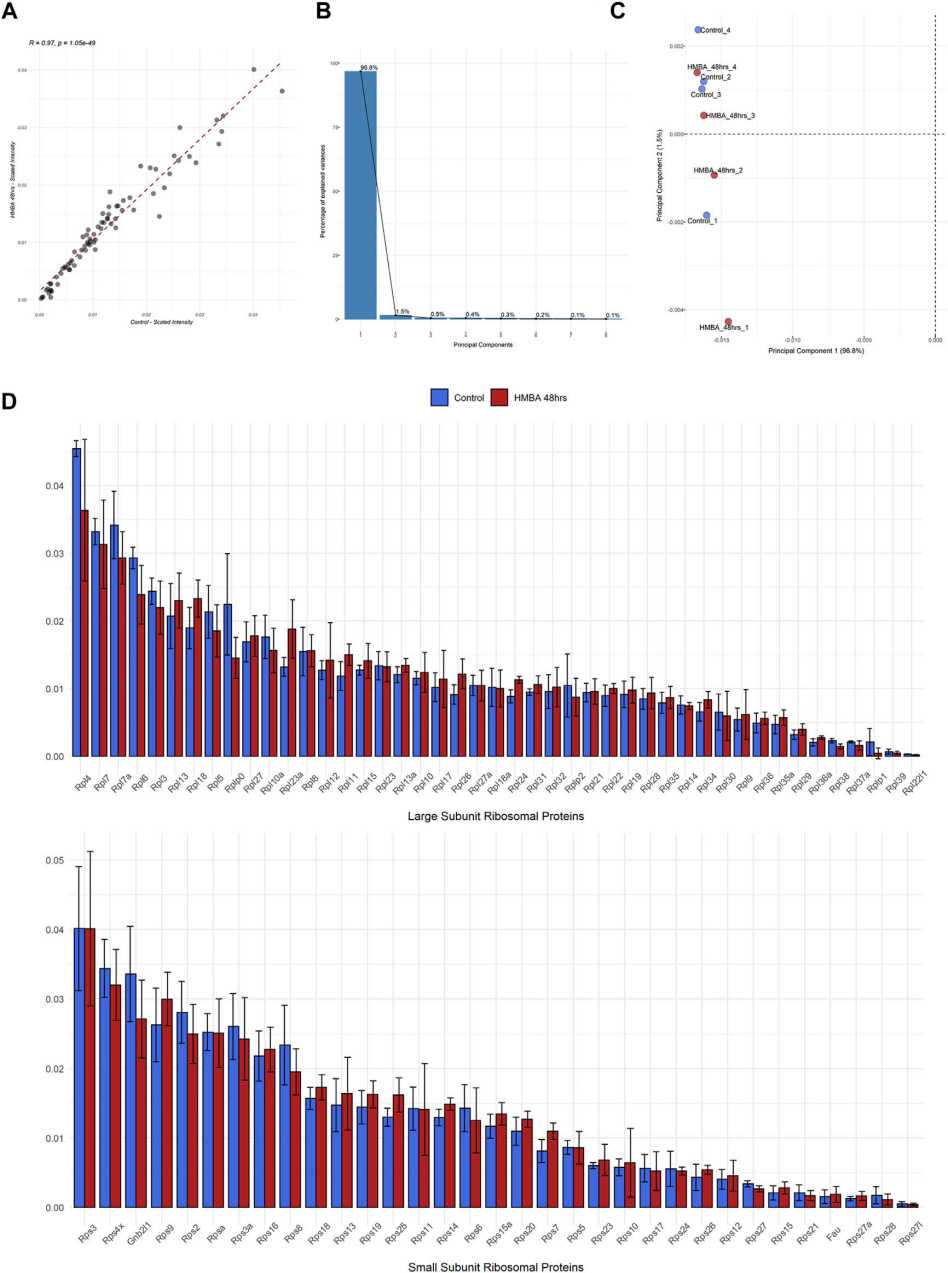
Proteomic analysis of the RP component of the ribosome during MEL cell differentiation. **(A)** Scatterplot of the average scaled intensity values per RP, depicting a strong positive correlation (Pearson correlation coefficient = 0.97) in RP stoichiometry between untreated (Control) and differentiated (HMBA 48 h) MEL cells. **(B)** Scree plot depicting the percentage of variance explained by each principal component (PC) for the analysis shown in C. **(C)** Scatterplot of Principal Components (1st and 2nd) of all independent biological replications of the ribo-proteomics experiment. Principal Component Analysis (PCA) was performed using RP scaled intensity values. **(D)** Bar plots depicting the average scaled intensity of each RP in Control and HMBA 48 h (upper panel large subunit RPs, bottom panel small subunit RPs). Error bars correspond to the standard deviation of scaled intensity values per RP and differentiation stage.

We then investigated the quantitative variations of RPs between the two samples by calculating the relative amount that each RP represents in the ribosome (the intensity of each RP was divided to the sum intensity of all the RPs, data analysis given in Supplementary Data Sheets S2–4). By this analysis, we found that not only the exact same set of 78 RPs was detected in all samples but, also, the independent RP stoichiometry values demonstrated excellent correlation (R = 0.97) between the samples (Figure 3A). Indeed, the RP expression levels of both subunits were invariable between the two samples and some minor variation was statistically insignificant (Figure 3C). This effect was, also, seen for the 2 RP paralogs (Rpl22l/eL22L1 and Rps27l/eS27L), despite that paralog genes frequently show differential regulation and tissue-specific expression. Moreover, we performed principal component analysis (PCA) of all the 8 samples that we analyzed (4 biological replicates for each of the two stages) using only the RP stoichiometry values. Importantly, all samples clustered together in the space of principal components 1 (PC1) and 2 (PC2), with the first explaining almost all the variation (96.8%) found in the dataset (Figure 3B). In conclusion, all samples are essentially identical in terms of RP stoichiometry suggesting against a model of ribosome heterogeneity in these sample types.

### Immunoblot Analysis of Selected RPs Validates the Mass Spectrometry Findings

To validate the findings generated through the MS/MS analysis, we assessed the expression of 6 selected RPs independently with immunoblot assay. Again, purified polysomal fractions were used to quantitatively determine RP expression using specific antibodies. Notably, similar to the proteomic analysis, all RPs tested, namely Rack1, Rps23, Rps6, Rpl4, Rps7 and Rpsa, showed no variation between the polysomal fractions of the independent stages of differentiation by this method (Supplementary Figure S4A, left panel). On the contrary, the levels of the same RPs showed a linear decline, as expected, when assessed on the whole cell level (Supplementary Figure S4, right panel). Thus, the polysomal RP composition and stoichiometry seem to remain constant during erythropoiesis even though the expression of most ribosomal constituents decreases in the whole cell level. Quantification of the immunoblot images, consistent with the proteomic data, revealed no alterations for the tested RPs in the polysomal fractions across differentiation (Supplementary Figure S4).

### Ribosomal Composition Remains Constant in Various Models of Erythropoiesis

**FIGURE 4:**
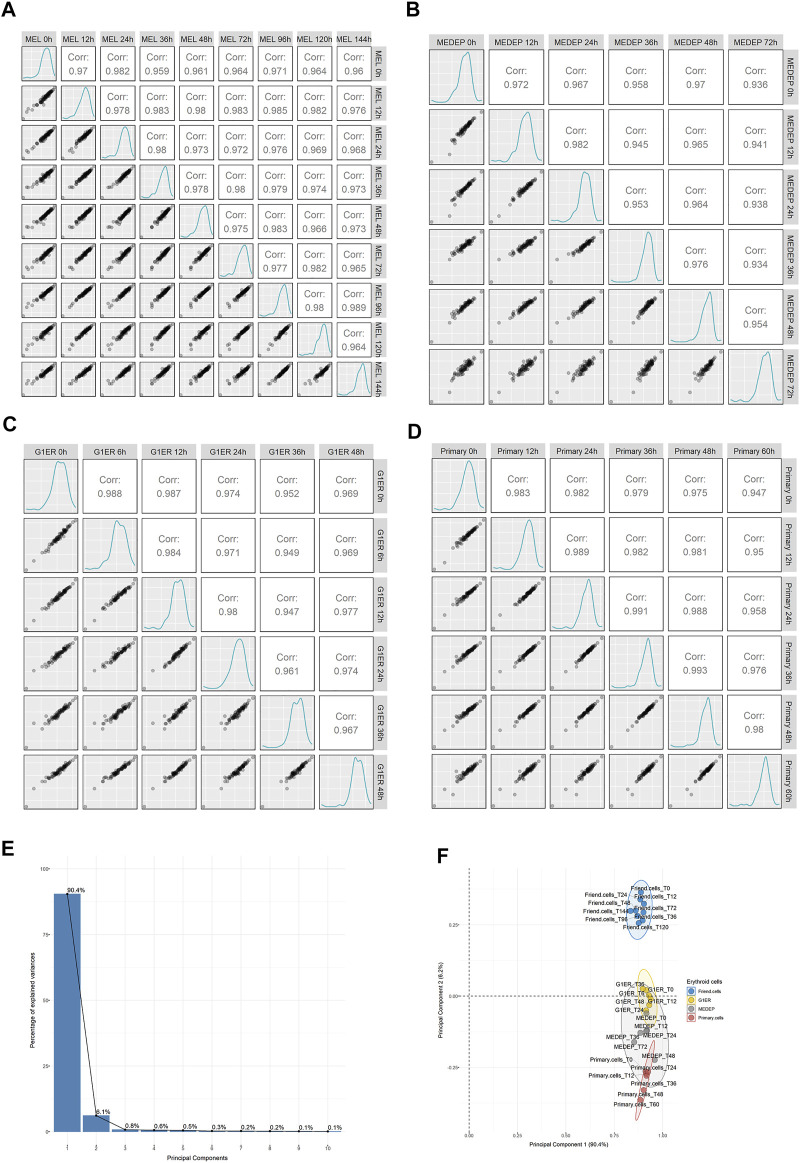
Regulation of ribosomal proteins on a whole cell level in proteomics data out of 4 models of murine erythroid differentiation (retrieved from Gautier et al.). Scatterplots depicting the relationship of average normalized intensity values of RPs across all stages of differentiation in: **(A)** MEL, **(B)** MEDEP, **(C)** G1ER and **(D)** primary cells. **(E,F)** Principal component analysis (PCA) using normalized intensity values of RPs for all independent samples from the study of Gautier et al. The scree plot of Panel E depicts the percentage of variance explained by each principal component (PC), and the scatterplot of Panel F shows the distribution of samples in the space of PC1 and PC2. Notably, variation is mainly explained by PC1 and PC2 while the independent samples form distinct clusters based on the cell type rather than the differentiation stage, thus supporting invariable ribosomal protein stoichiometry during erythroid differentiation.

Erythroid differentiation involves a precisely orchestrated program of proteome remodeling, characterized by a massive quantitative reduction of most proteins. We reasoned that if heterogeneous ribosomes played a role in erythropoiesis, then some RPs would be differentially regulated during the process of differentiation. To test this possibility, we retrieved published data from a whole cell proteomic analysis of MEL cells along with three additional models of erythropoiesis: MEDEP, G1ER and CD34 primary cells (Gautier et al., 2020). By extracting the expression values of 76 identified RPs and comparing them across all sample types, we found that independent RP expression demonstrated excellent correlation between the samples. This trend was seen in all cell types (Figure 4: MEL panel A, MEDEP panel B, G1ER panel C, primary cells panel D) even between the most differentiated cells against the undifferentiated cell types. Moreover, PCA was performed on all samples, using RP expression values, and distinct clusters based on cell type rather than differentiation stage were formed, denoting cell type as a more important source of variation for RPs during differentiation (Figures 4E,F). Thus, this analysis, again, supports that RPs are co-regulated during erythropoiesis and there seems to be no evidence for RPs heterogeneity in any of the differentiation stages.

## Discussion

Heterogeneity of the main ribosomal composition has been, recently, proposed as a mechanism of translational regulation in eukaryotes. Moreover, it has been suggested that mutations in RP genes that disrupt this type of heterogeneity may cause selective toxicity in patients with ribosomopathies (Narla and Ebert, 2010; Xue and Barna, 2012; Danilova and Gazda, 2015). To elucidate ribosome composition and regulation during erythroid differentiation we performed a detailed examination of the ribosomal population in MEL cells, along with a whole cell data analysis derived from 4 additional models of differentiation. Our data strongly supports that, while the ribosome population is re-organized the main ribosomal composition remains unaltered during differentiation.

Ribosome isolation in this work was performed using a sucrose gradient ultra-centrifugation protocol that enabled the quantitative assessment of all ribosomal subcomplexes (free ribosomal subunits, monosomes, polysomes) across the samples of interest. By this analysis, we found that ribosomes are subjected to strict quantitative regulation (decrease of polysomes and increase of monosomes) that begins with the onset of the MEL cell differentiation and is maintained through its final stages. As a consequence, the total amino acid incorporation and ribosome availability are likely very limited during late erythroid differentiation. Interestingly, it was previously shown that reduced ribosome levels can regulate which mRNAs will be preferentially translated, as ribosome reduction imposes a competition between the cytosolic mRNAs for the available ribosomes (Mills and Green, 2017). Intriguingly, selected mRNAs may be over-translated even under conditions of ribosome reduction owing to special *cis*-elements located in their 5’ untranslated region (Mills and Green, 2017). Thus, ribosome re-organization may prioritize which mRNAs should be translated and which should be inactivated during erythroid differentiation. This phenomenon likely represents a quick and effective mechanism of proteome remodeling that favors the continuous synthesis of selected proteins in a background of a widespread translational blockade.

Moreover, as stated above, our data suggests against the hypothesis of RP heterogeneity during erythropoiesis. Previous studies have, also, found a steady stoichiometry of RPs in mice tissues (Amirbeigiarab et al., 2019), prostate cancer cell lines (Reschke et al., 2013) and in a large-scale analysis across 33 human tissues (Kyritsis et al., 2020), thus questioning the hypothesis of ribosome heterogeneity for these cell types. In addition, the RP stoichiometry was found unaltered in hematopoietic stem cells with reduced levels of 3 genes associated with DBA development (RPS19, RPL5 and TSR2), albeit in this study the ribosomal architecture during differentiation was not assessed (Khajuria et al., 2018). Thus, by accurately quantifying the levels of 78 RPs during erythropoiesis, our data complements the study of Khajuria et al. (2018) and concurs that ribosomes remain stable in erythroid differentiation. This knowledge, if seen in a broader biological context, challenge the significance of RP heterogeneity and its associated ribosome specialization theory for gene expression regulation.

Nevertheless, our study has certain limitations. Firstly, we cannot exclude that additional mechanisms of heterogeneity, such as differential rRNA modifications, alterations of ribosome associated proteins and post-translation modifications, may play regulatory roles during differentiation. A more detailed analysis of the ribosomal population towards these directions will greatly advance our understanding of ribosome function in health and disease. Moreover, as we have only assessed the polysomal fraction of ribosomes there is the possibility that alterations may exist in other ribosomes subtypes such as the monosomes or the free subunits. However, these ribosomal types are largely passive and merely engaged in translation, therefore undermining the biological significance behind such a phenomenon. In any case, future studies that would study ribosome composition across additional models of erythroid differentiation as well as between healthy and leukemic cells are of great interest in the field.

Finally, our data is in line with the current view of understanding concerning the mechanism of toxicity in patients with DBA. Indeed, it is believed that haploinsufficiency of selected ribosomal proteins primarily provokes a reduction of the overall ribosomal levels in patient cells (Mills and Green, 2017). This ribosomal shortage is added to the physical reduction that happens due to erythroid differentiation (also shown in this study, Figure 1), therefore leading to toxicity. Nevertheless, while some proteins, including the critical erythroid factors GATA-1, BAG1, CSDE1, are heavily affected by the presence of special 5′UTRs that render them poor ribosome recruiters, main regulators of tissue development in most of the other tissues remain unaffected. Hence, patients are unable to synthesize critical erythroid promoters and suffer from anemia but do not demonstrate general toxicity in other organs. In conclusion, the robust proteomic architecture of ribosomes implies that therapeutic strategies for this disease should be directed towards the main ribosomal pool and not any particular heterogeneous population.

## Data Availability

The original contributions presented in the study are included in the article/Supplementary Material, further inquiries can be directed to the corresponding author.
