# Supplementary material for: Invariable Ribosome Stoichiometry During Murine Erythroid Differentiation: Implications for Understanding Ribosomopathies
Source: Front Mol Biosci. 2022 Feb 3;9:805541. doi: 10.3389/fmolb.2022.805541 (PMC8850788; doi:10.3389/fmolb.2022.805541)
Supplement: Supplementary file 3 [file DataSheet1.docx]

Supplementary Material

## Supplementary Figures

##
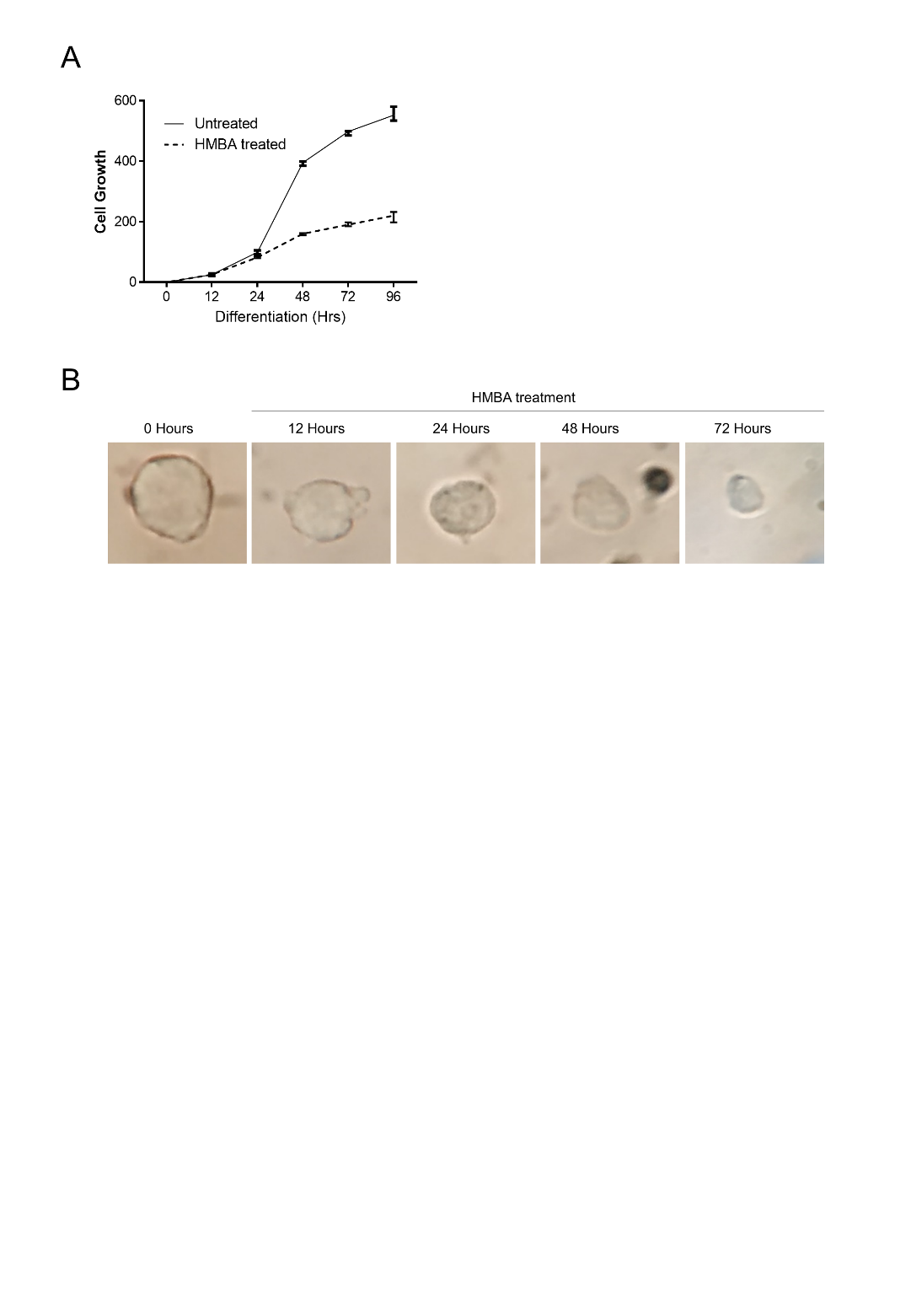


## Supplementary Figure 1. MEL cells as a model of erythroleukemia differentiation. (A) Cell proliferation of MEL cells treated with HMBA (5 mM) in comparison to control untreated cultures (B) Differentiation-dependent decrease in cell size of MEL cultures observed under the optical microscope at a 100x magnification.

**
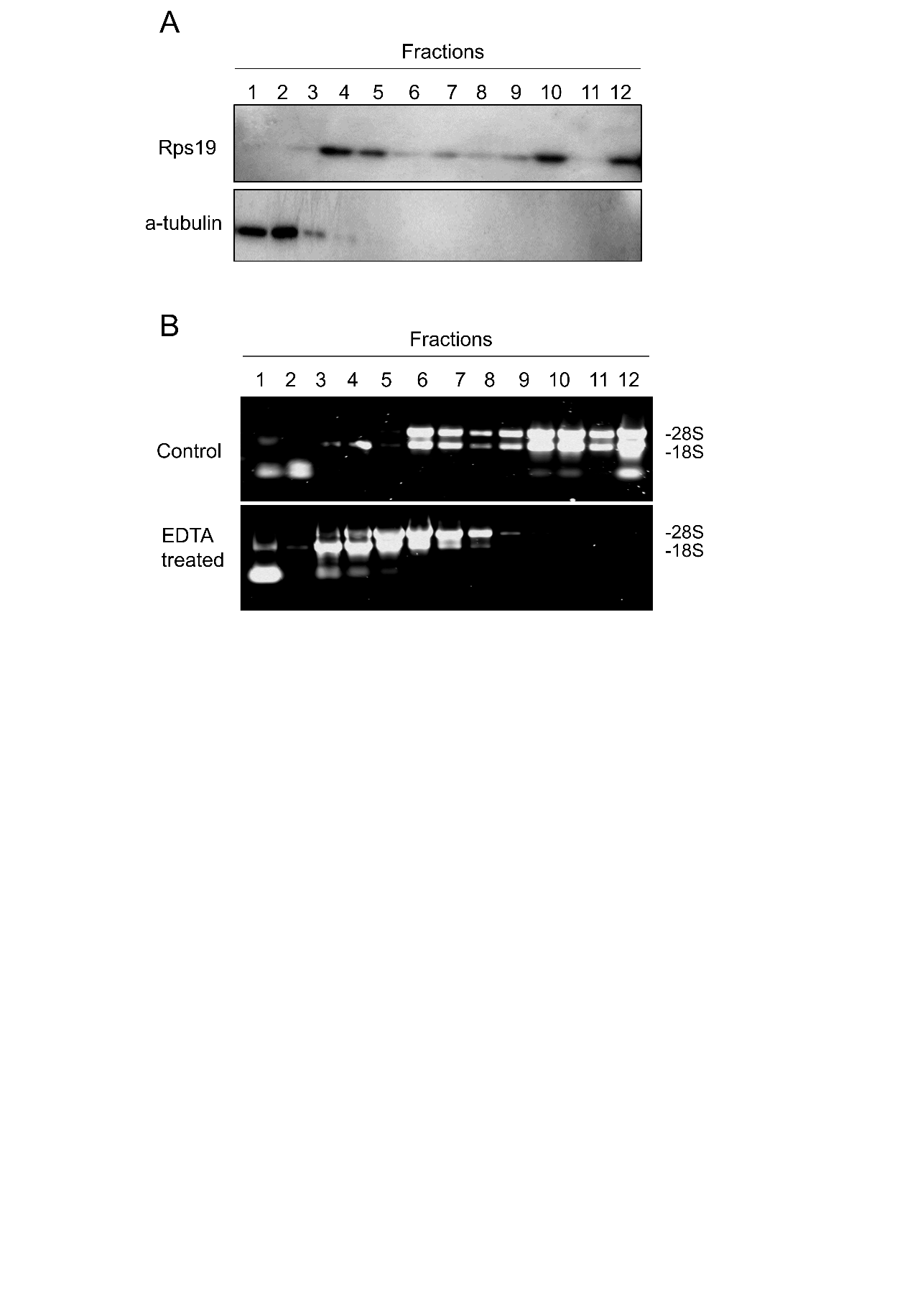
**

**Supplementary Figure 2.** Ribosome fractionation in MEL cells. (A) Western blot analysis using separated fractions (12) from control MEL cells, with a ribosomal marker (Rps19) and a cytosolic marker (b-Tubulin). (B) Same as with A but here RNA analysis by electrophoresis in an agarose gel was conducted. EDTA (50mM) treatment results in subunit dissociation, thus this sample represents a control for the efficiency of ribosome fractionation.

**
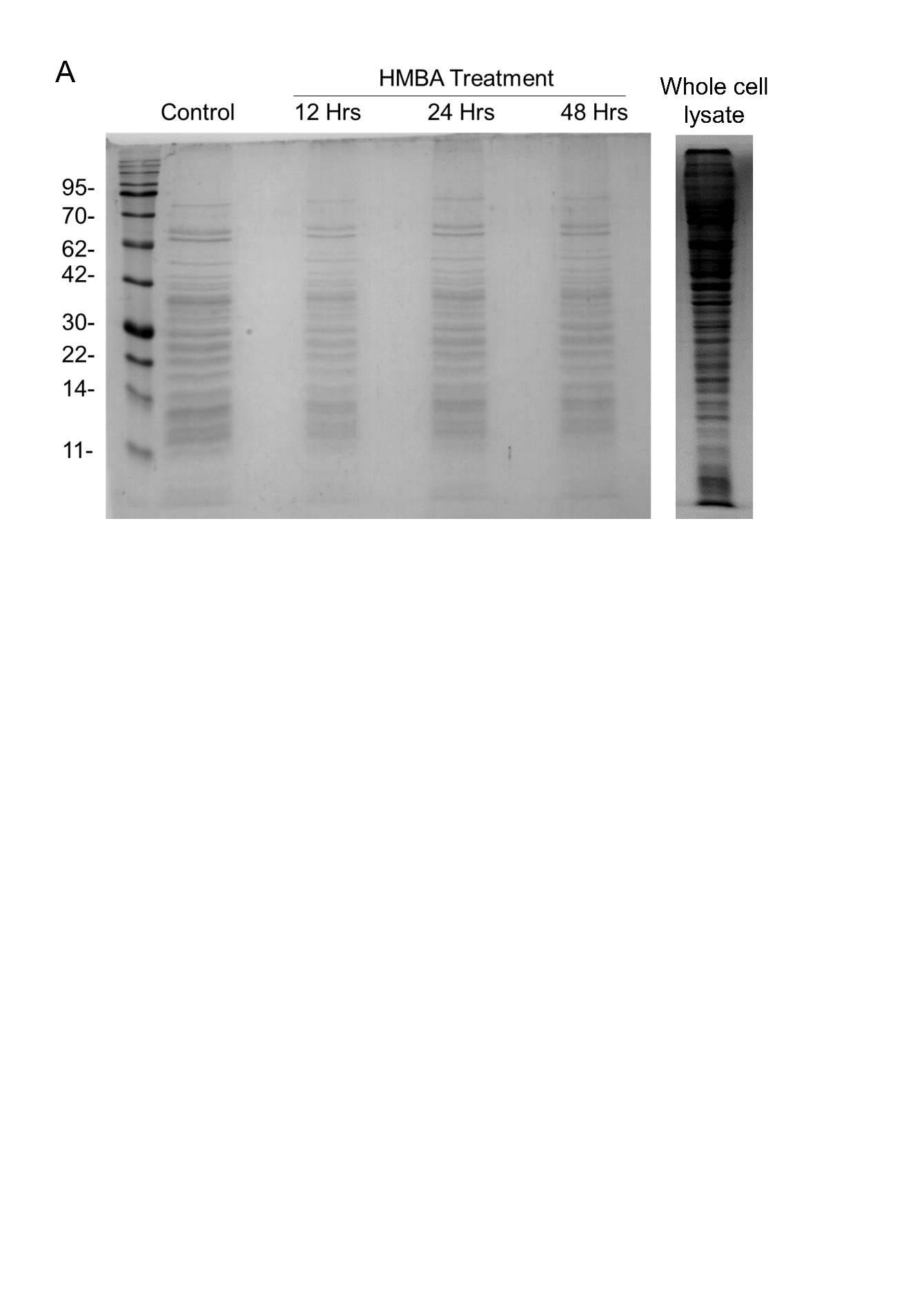
**
**Supplementary Figure 3**. Analysis of polysomal proteins across 4 stages of MEL cell differentiation. Proteins were resolved through electrophoresis in an SDS-PAGE gel (14%) and visualized with blue silver staining. Polysomal samples are shown next whole cell lysates.


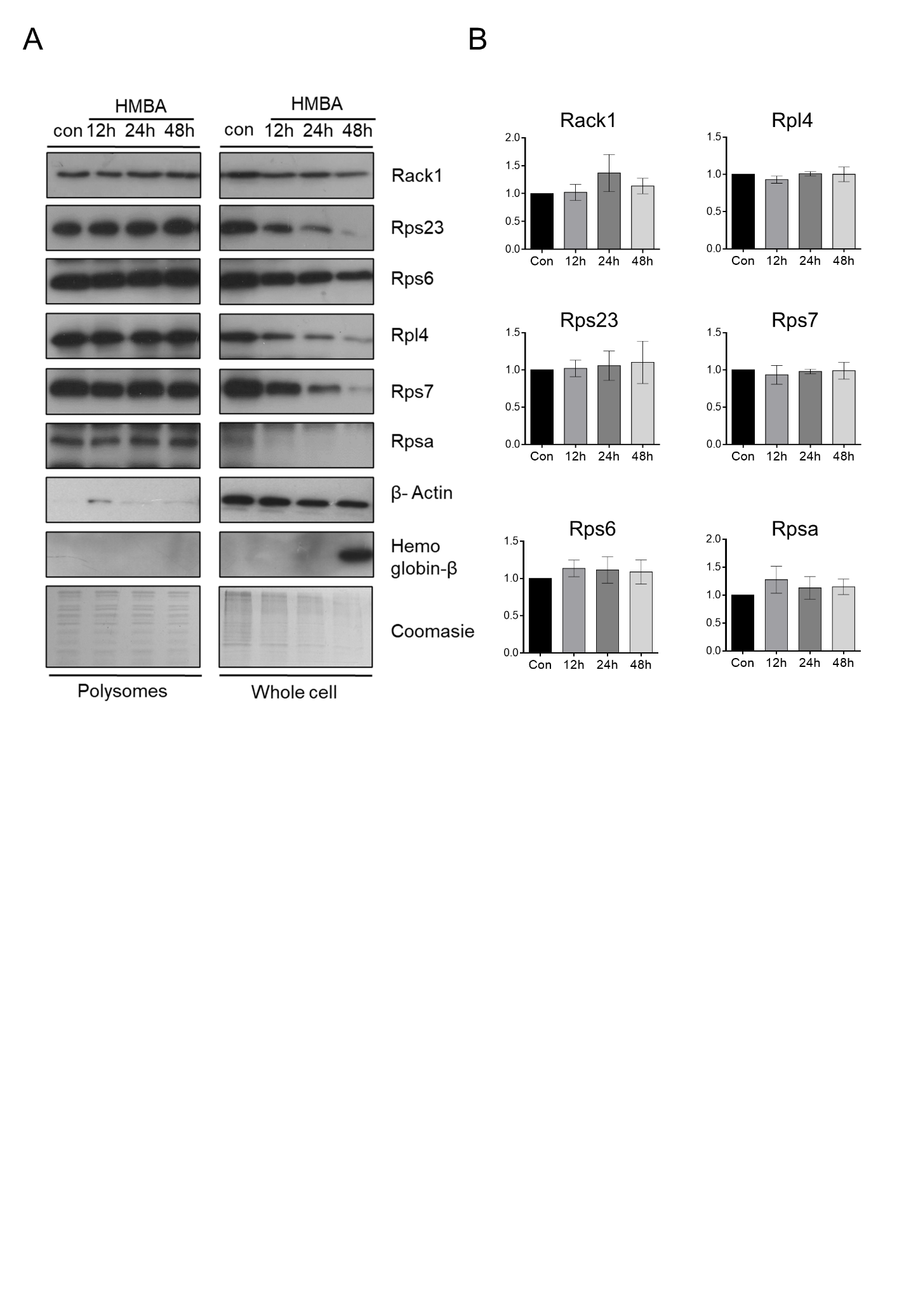


**Supplementary Figure 4**. Quantitative analysis of selected RPs by western blot. A) Antibody mediated detection of the indicated proteins in isolated polysomes as well as in whole cell extracts (experiment was repeated 3 times). B) Quantification of the polysomal bands from the western blot images. The value of each RP was normalized to the Coomassie staining value of the corresponding sample.
