# Supplementary figures and images for: Invariable Ribosome Stoichiometry During Murine Erythroid Differentiation: Implications for Understanding Ribosomopathies
Source: Front Mol Biosci. 2022 Feb 3;9:805541. doi: 10.3389/fmolb.2022.805541 (PMC8850788; doi:10.3389/fmolb.2022.805541)

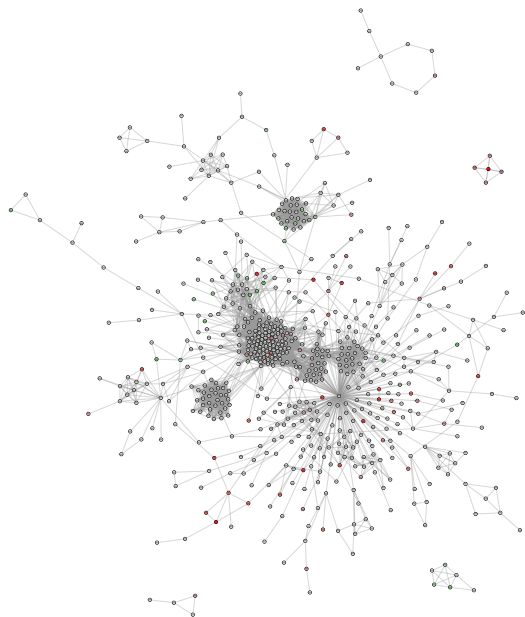

Supplement: Supplementary file 4 [file Image1.pdf]
